# Health and economic implications of the ongoing coronavirus disease (COVID-19) pandemic on women and children in Africa

**DOI:** 10.1186/s12978-023-01616-w

**Published:** 2023-05-08

**Authors:** Helena Yeboah, Sanni Yaya

**Affiliations:** 1 School of International Development and Global Studies, Faculty of Social Sciences, University of Ottawa, Ottawa, Canada; 2 The George Institute for Global Health, Imperial College London, London, UK

**Keywords:** COVID-19 pandemic, Maternal and child health utilization, Nutrition, Domestic violence, Africa

## Abstract

The coronavirus disease (COVID-19) pandemic continues to pose major health and economic challenges for many countries worldwide. Particularly for countries in the African region, the existing precarious health status resulting from weak health systems have made the impact of the pandemic direr. Although the number of the COVID-19 infections in Africa cannot be compared to that of Europe and other parts of the world, the economic and health ramifications cannot be overstated. Significant impacts of the lockdowns during the onset of the pandemic caused disruptions in the food supply chain, and significant declines in income which decreased the affordability and consumption of healthy diets among the poor and most vulnerable. Access and utilization of essential healthcare services by women and children were also limited because of diversion of resources at the onset of the pandemic, limited healthcare capacity, fear of infection and financial constraint. The rate of domestic violence against children and women also increased, which further deepened the inequalities among these groups. While all African countries are out of lockdown, the pandemic and its consequent impacts on the health and socio-economic well-being of women and children persist. This commentary discusses the health and economic impact of the ongoing pandemic on women and children in Africa, to understand the intersectional gendered implications within socio-economic and health systems and to highlight the need for a more gender-based approach in response to the consequences of the pandemic in the Africa region.

## Introduction

The novel coronavirus disease (COVID-19) pandemic, which began in December 2019 has been marked as the global health crisis of today’s society with more than 670 million infections and over five million related deaths across 227 countries and territories [1]. As of August 4, 2022, 55 African countries had recorded a total of more than 11.9 million infections and over 255 thousand deaths [2]. Although the aggregate COVID-19 cases in Africa are incomparable to that of Europe and other parts of the world, the economic and health ramifications cannot be overstated. About 85% of Africa’s trade is with the rest of the world and therefore lockdowns in China, Europe and the United States resulted in a decline in the demand for African exports, imports of capital and consumer goods, tourism, and foreign direct investment (FDI) inflows [3, 4]. The consequences on Africa’s economies were more pronounced at the onset of the pandemic given the extent of the decline in global demand and financial inflow [5].

While all African countries are out of lockdown and other restrictive measures, the pandemic and its consequent impacts on the health and socio-economic well-being of women and children persist. This commentary therefore discusses the impact of the ongoing pandemic on the health and wellbeing of women and children in Africa, particularly in the areas of reproductive health access and utilization, gender-based violence, forced/early marriages, and food insecurity and nutrition in two time periods, the first being during the COVID-19 pandemic lockdown, and the second, after the COVID-19 pandemic lockdown. This is to understand the intersectional gendered implications within socio-economic and health systems and to highlight the need for a more gender-based approach in response to the consequences of the pandemic in the Africa region.

## COVID-19 pandemic lockdown and the health and economic impacts on women and children in Africa

In response to the spread of the pandemic, most governments instituted preventive measures including lockdowns, movement restrictions and social distancing, which resulted in the closure of certain businesses, airports, schools, and vital social services. The resulting consequences were job and income losses, as a higher percentage of people in African countries rely on informal work [6]. Within the first month of the COVID-19 crisis, the International Labor Organization [7] estimated a decline in revenue of about 81% for Africa’s informal workers. However, women and men experienced the pandemic and the impact of these measures differently. Evidence showed that out of the total proportion of individuals in the informal sector, approximately 70% were women, who dominated small enterprises like the sale of cooked food, vegetables, foodstuffs and second-hand clothes at the market, which implied that such a large number of women had little to no income security, health insurance or social protection [8]. Moreover, since they earned and saved less, they became highly susceptible to economic shocks.

### Health implications of the pandemic on women and children

The COVID-19 pandemic and lockdown measures did put pressure on both the demand and supply of healthcare and health systems globally. Besides revealing the gaps and inadequacies in Africa’s health systems, the pandemic restriction exacerbated serious societal and health problems as school closures and lockdowns disrupted access to essential health services, increased gender-based violence and early marriages, which negatively affected reproductive health and overall wellbeing.

#### Health services access and utilization

At the onset of the pandemic, the fight against the spread of the virus required the mobilization of resources to acquire ventilators and personal protective equipment (PPEs) such as gloves, masks, and overalls for frontline workers. This meant that resources were diverted from critical routine health services, particularly those related to sexual and reproductive health services for women, to managing the COVID-19 pandemic. Consequently, the risks of maternal mortality and morbidity, HIV/AIDS infections and related deaths, adolescent pregnancies, and sexually transmitted diseases were heightened [9, 10]. Other factors such as constraints of resources, particularly logistical support for healthcare providers, also contributed to declines in maternal and child health service utilization including family planning, antenatal care, and immunization [11]. Delayed vaccination campaigns and low routine immunization coverage in many countries resulting from resource demand of the pandemic contributed to 7.7 million children in Africa missing the first dose of Diphtheria-Tetanus-Pertussis (DTP–1), representing 45% of the global Fig. [12]. Consequently, in 2022, 14 out of 23 countries in Southern and Eastern Africa had experienced major measles, cholera, and poliovirus outbreaks [13].

In Uganda, facility-based deliveries declined by 3% and maternal deaths also increased by 7.6% [14]. Other affected maternal health outcomes included antenatal, sexual, and reproductive health, emergency, obstetric, and postnatal care services [14]. Significant declines in facility-based deliveries and first antenatal care attendance were recorded in Sierra Leone and Liberia [15]. Women also avoided seeking health care due to the fear of healthcare-associated infections, which prevented them from assessing routinely required antenatal care (ANC) and post-natal health services [9, 16]. Significant decrease in neonatal hospital admissions and increase in neonatal mortality as a result of combination of factors including limited healthcare capacity, financial constraint, and fear were reported in the Northern part of Ghana [17]. In South Africa, a study found significant decline in the number of women who utilized the services of healthcare providers, but not among men [18]. However, for women with no post-secondary education, the gender gap was more evident.

While many studies confirmed the above adverse impacts of the pandemic on the health of women and children, other reports showed that utilization of maternal and child health services varied across different countries, maternal health outcomes and waves of the pandemic [19]. The level of impact of the pandemic in a particular country could be influenced by the extent of the outbreak, the influence of restrictive mitigation measures and the preparedness of the health system [19]. Another research pointed out that as the pandemic progressed, it had to be recognized that the impact was not homogeneous among low-middle income countries or even within the same country [20, 21]. Findings were that at the onset of the pandemic, certain sub-group of the population, specifically, young women were more affected, compared to older women when it came to access to sexual reproductive health, particularly contraceptive commodities [20, 21].

Another study revealed that in one of Ethiopia’s referral hospitals, while the number of hospital deliveries was stable, family planning programs declined by 95%, antenatal care decreased by over 50% and neonatal admissions, including child emergency visits also decreased by over 70% [22]. Findings from six referral hospitals in Guinea, Nigeria, Tanzania, and Uganda also showed that despite few interruptions during the first wave, the provision of routine maternal care was maintained [23]. In the Democratic Republic of Congo, even though the rates of facility-based childbirth and second post-natal care visits were not significantly impacted by the pandemic, ANC decreased by almost 45%, following the start of the pandemic in Kinshasa [24]. Also, in Kenya, women continued to access health facilities, although, about 40% of study respondents reported declined access because of fear of being infected [25].

#### Domestic violence and early marriages

Domestic violence is a multifaceted phenomenon grounded in a throng of factors including situational factors, and therefore tends to thrive in contexts where vulnerabilities persist [26]. It is therefore not surprising that in Africa, intimate sexual violence is widespread, standing at 36%, and exceeding the global average of 30% [27]. Prior to the pandemic, data showed that in over 80 countries, one in three women experienced physical and/or sexual based violence by an intimate partner in a relationship at some point in their lives [28]. Moreover, evidence had shown that in the context of crises, women endured compounding forms of violence [29].

The COVID-19 pandemic nonetheless disrupted economic activities, increased unemployment, and declined incomes, resources, and access to social support and safety nets (24—29). This created a conducive environment for perpetrators and triggered or further compounded the risk of domestic and/or intimate partner violence. Indeed, the surge in domestic violence worldwide resulting from the pandemic earned it the term “intimate terrorism” [30]. The increase in reports of domestic violence moreover occurred concurrently in a period where many countries were experiencing compromised healthcare services [31]. Life-saving support, resources and care given to women who experienced domestic or intimate partner violence were totally disrupted or less accessible [31]. In many areas in Africa, such support was not encouraging even in the pre-COVID-19 era, which meant heightened risk of violence and debilitating health impacts.

Other social problems that increased in many African countries were the rates of child, early and forced marriages and adolescent pregnancies [32]. Pre-pandemic estimates showed that 30% of girls in 30 African countries experienced child or forced marriages, with as many as 15 million girls being married off before age 18 [33]. In four Eastern and Southern African countries, Ethiopia, Mozambique, Uganda and Zambia, where the Global Programme to End Child Marriage (GPECM) was being implemented, data showed that adolescent girls were severely impacted by the pandemic as the rates of violence, child marriage, and teenage pregnancies increased [32]. The rise in these numbers were partially attributed to school closures, and limited access to sexual and reproductive health services. In a household survey in Kenya, Uganda, Ethiopia and Senegal, findings showed that the COVID-19 pandemic contributed to perceived increase in child and forced marriages in Kenya because of school closures and loss of income [34]. However, the authors found that in Ethiopia and Senegal, the pandemic had limited perceived effect on child or forced marriages, and minimal perceived effect on child and forced marriages in Uganda.

### Economic implications of the pandemic on women and children

Beyond the numerous negative health impacts, the pandemic also caused deep economic damage in many African economies. The level of global interconnection and interdependence Africa has with Europe and other parts of the world sent waves of economic shocks across many African countries [35]. This resulted in declines in growth rates, with further estimation of a declined growth for Africa from 4.1% in 2022 to 3.8% in 2023. While the rate of poverty has been persistently and historically high in the region, COVID-19-induced outcomes such as a significant rise in price levels due to disruption in supply chains, falling growth in per capita income and high external debt, have kept many economies and vulnerable population in poverty [35].

One of these shocks was the increase in food insecurity and undernutrition. Prior to the COVID-19 pandemic, Africa recorded the highest prevalence of undernutrition in all of its sub-regions and was home to over 250 undernourished people in 2019. This made it extremely impossible for the continent to advance the achievement of the SDG 2 which calls for an end to hunger, and achieve food security, while improving nutrition and promoting sustainable agriculture [36]. Unfortunately, the COVID-19 pandemic posed a significant threat to both the demand and supply sides of the global food systems. As reported in Sierra Leone during the Ebola outbreak, pandemics tend to disrupt agricultural production and food storage, processing, distribution, transport, trade, and retailing [37].

In Nigeria, transitory food insecurity occurred during the lockdown days, resulting from a combination of factors such as loss of household incomes and declined purchasing power, increase in post-harvest losses at both farm and market levels, increased food prices, increase in transportation costs and hoarding by marketers, and this led to significant impact on the population [38]. The estimation of the cost of a healthy diet in 2020 exceeded the international poverty line in Africa, with such healthy diets becoming five times more expensive than starchy staples that met only energy needs, and this made healthy food unaffordable to the poor [39]. Consequently, in 2021, almost 25 million people in the region were unable to meet their food needs, representing 34% higher than in 2020 [40].

Research has shown that women and children are disproportionately impacted during economic crises, especially when it comes to food security and nutrition [41]. Often, women and girls are vulnerable on all dimensions of food security, and they suffer nutrient deficiencies. Being the main caretakers of children, the elderly and the sick, women tend to restrict their food consumption in their efforts to make more available for the rest of the household [42-44]. With high food prices, the affordability and consumption of healthy diets among this vulnerable group became more difficult [45]. For school children, school closures which occurred earlier on during the pandemic meant that children who relied on school meals lost such access, which contributed to poor feeding practices and micronutrient deficiencies [46].

## After COVID-19 pandemic lockdowns

Although lockdowns have been lifted in all SSA countries, the negative impacts on the health and socio-economic wellbeing of women and children linger on. The disruption of the pandemic to sexual, reproductive, maternal, newborn, child and adolescent health services, gender-based violence and early marriages will persist in many African countries for a long period of time. Moreover, research shows that while men are able to return to the pre-crisis level of living conditions within a short period, it takes much longer for women to return to pre-crisis levels of economic security and overall livelihoods [47].

Not only have the impacts of the pandemic further deepened persisting inequalities mainly along gender lines, but also, many women who did not access routine reproductive health services due to health system or personal resource constraint or fear, as recorded in several countries and studies, may certainly face other lasting negative effects [48]. For instance, reproductive health services such as antenatal and postnatal care are important in detecting pregnancy and after birth complications, resulting in positive birth outcomes and reduction in maternal and child mortality and malnutrition [49]. In the same vein, children who missed immunization doses and are not able to catch up, may lose protection against vaccine-preventable diseases, including cholera, measles, rotavirus, rubella and pneumococcal diseases, which affect over 30 million children in Africa yearly, out of which half a million die annually [12]. This translates that the obstruction of health access and utilization of these services due to the pandemic lockdown can be risk factors to complicated morbidities or mortalities in future.

Women and children who experience domestic violence encounter life-long physical, mental, psychological, or emotional traumas. Studies show that children who do not experience violence themselves, but who even witness these forms of violence in the household are highly likely to face similar negative issues as children who are victims of physical abuse, emotional abuse or neglect [50]. These impacts tend to influence deviant behaviors such as substance use and abuse, antisocial behaviours towards other children, and an increased likelihood of experiencing violence in their adult lives [50]. Furthermore, violence against women and girls can lead to negative economic related issues, in that, an increase in rates of violence against this group diminish economic activities and economic development of a country [51]. The mental and psychological traumas they experience can induce absenteeism and presenteeism which impact productivity and GDP growth. The authors add that in countries where there are no protective laws against domestic violence, the economic cost of violence against women is high, and this poses a threat to economic development.

Currently, all schools in African countries that went on lockdowns are reopened, which means that school feeding programs in countries where they existed during the pre-COVID period may have been resumed. However, if children are deprived of vital nutrients over time, they become malnourished, which leads to the development of chronic health issues as immune systems weaken [52]. Similarly, with the reopening of schools, reproductive health and other vital programs that engage girls to stay in school may have resumed their operations to provide essential resources and services, however, children who were married off during the lockdown period may not have any opportunity of returning to school, which may deprive them of economic independence in their adult lives. Moreover, child marriages have significant long-lasting negative impacts on the overall wellbeing of these girls. There is the high likelihood for girls who marry to experience domestic violence, face unplanned pregnancies, increase risks of pregnancy complications, and maternal and infant mortality [53]. Overall, it’s been projected that lost schooling will have longer term impacts on children, with such effects being disproportionately high for children with lower socio-economic backgrounds [54].

The recovery from the pandemic may vary across the African sub-regions, with some parts faring better quickly than others, however, with the fall in global demand, global inflation, and higher borrowing costs, among other factors, the continent is far from full recovery from the pandemic [35].

## Conclusion

The COVID-19 pandemic has evidently had negative consequences on global health and economies, particularly, in countries in Africa persistently plagued with weak healthcare systems and deprivable maternal and child health outcomes. It is therefore fair to expect a more grievous long-term impact on the health, socio-economic and overall wellbeing of women and children in Africa. Generally, there are still ongoing impacts of long-COVID, which will require further mobilization of resources, and this will likely put a strain on already weak health and economic systems [54].

Although lockdowns have been lifted in all African countries, the disruptive impact of the pandemic restrictions on maternal and child health outcomes will still linger on. While these impacts may be overwhelmingly innumerable and intense, it is not too late to provide comprehensive and tailored services in schools and local communities to address these complex issues through a more gendered lens by prioritizing women’s safety in African countries.

As governments and policymakers in the region allocate resources to address the COVID-19 pandemic, comprehensive programs focusing on sexual and reproductive health, continuous education and awareness creation, social welfare services, and research should be part of national and institutional budgets. This will equip the  most vulnerable population to deal with the negative impacts of the pandemic. In individual countries, existing policies geared at protecting the rights of women and children against domestic violence and forced marriages should be purposefully enforced, as these factors have multi-throng effects on the overall health of these individuals as well as the economy, both in the short and long run. Without the implementation of strategic interventions, these numbers will rise exponentially by 2050 (33). Significant measures should also be pursued remembering the fact that the improvement in women’s health is a measure of progress and development in countries.

In learning from the ongoing COVID-19 pandemic, African countries need to remain alert and have action plans in place to effectively deal with future crises. This will help build the capacity of the health and economic systems to withstand emergencies while sustaining the provision and utilization of key services. The pandemic has had intersectional gendered implications and therefore there is a need for a more gender-based approach and response to the continuous impact on women and children in African countries.

## Data Availability

Not applicable.
